# St. Louis Obstetrical and Gynecological Society

**Published:** 1880-03

**Authors:** 


### Reports of Societies.

### ST. LOUIS OBSTETRICAL AND GYNECOLOGICAL SOCIETY.

*Albuminuric Convulsions*—Dr. Prewitt: I was called the evening before last to see a lady in the seventh month of pregnancy, whom I found with swollen feet, pale face, and suffering from the most intense headache, with a pulse that was rather hard, and had considerable tension, and with some neausea. I apprehended convulsions, because I was satisfied from the general symptoms that there was albuminuria, and I prescribed for her. Her husband was not at home at the time. I left with the request, if she was no better in two or three hours, to let me know. That was about seven o'clock in the evening. Between 10 and 11 P.M., a messenger came, saying she had a convulsion. When I got to the house, I found her in a state of semi-consciousness, not being able to recognize me. I prescribed active cathartics, etc. She passed through the night without another convulsion. On examing the urine yesterday morning, I found it perfectly solid on heating it, so that when the test tube was turned upside down, not a drop was lost, and I had difficulty in washing it out with a stream of water, so solid was it with the albumen. The amount of urine which she lost seems to be not very far short of the usual quantity. I learned from her that she had been having swelling about her feet for the last two weeks, but during the preceding two days, it had greatly increased, as had the paleness about the face. I saw her this morning, and again this evening; there was less puffiness, less swelling, and the headache had subsided. She spoke a little and looks better, but it is only the seventh month of pregnancy, and she will have to run the gauntlet for the next two months in that condition of things. Now the question with me is, whether she is likely to do it in safety, or whether the probability is not that I will have to produce premature labor in order to save her life. With a convulsion already, with the urine loaded with albumen, and the other symptoms, I never saw a case that I felt more anxiety about.

Dr. Coles: Did you test the urine before she had convulsion?

Dr. Prewitt: No, sir, I had no opportunity of doing so. I think I examined her urine some time before she became pregnant. She was then suffering from irritability of the bladder, and I prescribed for her without finding albumen at the time. There was little, if any, cystitis.

Dr. Boisliniere: In a case like this, I would not interrupt the labor, but would bleed the woman, following the teachings of the immortal Dewees and Meigs. You may talk as much as you please, but you will have to use the lancet. When a woman has this intense headache, dimness of vision, etc., these changes are significant; and, with the pulse tense, the urine scanty, etc., I would not hesitate to bleed. I would not produce premature labor, because the attempt might produce convulsions. I think it is a pretty well established rule, that unless the indication is very great, it is not well to interfere in these cases, unless the cervix be dilated. Wait until this takes place, and then aid labor. I think the safest thing Dr. Prewitt can do in this case, is to follow in the old beaten track, and give this woman a good bleeding from the arm, and follow by saline purgatives, getting, in this way, a good deal of water from the blood, and so relieve the tension on the brain; and I think he will have a good success. You may talk, as is the fashion now among some, about the nervous origin of eclampsia, but here is a case that goes to disprove this theory, because, as far as I can see, there is no such cause of irritation in this case. Eclampsia has been compared to the irritation in teething children, but nothing of that kind exists in this case. She is albuminuric, but albuminuria exists in ninety per cent. of cases of convulsions. Two or three days ago I was called to see a woman—a primipara—who presented the same history, and who, after having several of these convulsions, had been bled. Dr. Papin had bled the woman twenty ounces, and I bled her twenty ounces more, and that put an end to the convulsions. If a woman, having these convulsions, has been bled, and the convulsions still continue, it is because she has not been bled enough. And what is the difference if a woman lose twenty or thirty ounces, or forty or sixty ounces of blood, who loses forty ounces of blood every month of her life?

Dr. Bauduy: In reply to Dr. Boisliniere's allusion to the neuropathic theory, I will simply say, that in my paper, I made no reference to the treatment at all, or at least only touched upon it.

Dr. Boisliniere: Because you were afraid to.

Dr. Buduy: Not at all; but the subject was too extensive. I spoke, however, of cases in which venesection would be a very proper remedy.

Dr. S. G. Moses: I recollect some years ago a lady who was on a visit to my family, who was suddenly seized with convulsions. I bled her very freely. I do not know how much blood I took, but enough to relieve her headache, and she never had another convulsion, but went to full term, and was delivered of her child. In cases of that kind there is more or less congestion of the kidney, producing albuminuria. I coincide with the doctor, as regards the practice, as far as my experience goes.

Dr. Maury (of Memphis:) I should heartily endorse the practice of bleeding.

Dr. S. G. Moses: I was on a race course once, and a man there who was suffering a great deal from headache, asked me to bleed him. I took about forty ounces of blood from him and he was immediately relieved; and in half an hour afterwards he came down and ate a hearty race dinner and drank champagne *ad libitum*, and without any return of the headache. During his life he was bled about once every four or five months. The man was the Prince Murat, who died a short time since in Paris, aged eighty. Dewees laid it down as an absolute necessity, that if a patient complained during labor of headache to bleed immediately. I do not know how it is that we are now so afraid to relieve our patients by venesection. I have never yet seen any bad effects from it. The sister-in-law of this gentleman whom I just mentioned, was bled on an average once a month, for twenty-five years of her life, and is still living, now about seventy years old. So that it would seem that bleeding will not kill, although there certainly are cases of disease in which it is unadvisable.

Dr. Prewitt: I was going to say that I looked up my lancet, and if there had been any further convulsion I should have used it, but she had no indication of further convulsion, and I thought I would wait. I do not think Dr. Boisliniere caught my idea exactly with regard to the production of premature labor. But I will say that as regards the bleeding, I fully concur in the view that it is important in these cases to bleed, and I propose to do it in this case if there are any further threatenings of convulsions. But the point I made was this: she is to go two months yet, with the kidneys and the general system in this condition, and can you hope by bleeding her, and the use of purgatives, etc., to so remove the condition as to get her safely through the next two months? The kidneys are undoubtedly congested. I examined the urine microscopically, and found one or two casts, but I take it that in almost all cases in which we have albumen in the urine, there is a certain amount of congestion; in fact, they are almost always in proportion.

Dr. Barrett: I do not see how it is possible for any one to determine whether it may be necessary to produce premature labor in this case or not. In New York City, all these kidney troubles are much more prevalent than here; you can go into a hospital there and pick out the patients suffering from these complaints, by means of their waxy faces, across the ward. In all the Eastern hospitals, it is the practice to put off these convulsions by the administration of cathartics, and the hot air bath. This is done by putting the patient in bed, and introducing hot air under the bed clothes; a very copious perspiration can be produced that way, and I have seen some of the most threatening cases go through without any difficulty, and even without convulsions. Jaborandi has also been lauded of late for that purpose, but I believe jaborandi is an oxytoxic. I think in Dr. Prewitt's case, I would give this woman, daily, perhaps some active saline cathartic. If it were necessary, I would also give a hot air bath daily, and await developments. If the case became so urgent as to make other treatment necessary, or very advantageous, I should resort to the induction of labor.

Dr. G. A. Moses: In the commencement of the evening, Dr. Maughs remarked that he thought the subject was exhausted. I do not think it has been more than touched upon. Certainly, the two papers (my own and Dr. Bauduy's,) did not touch the treatment at all; I purposely avoided it. I think each of these cases presents its individual aspects. It is almost impossible to have a set rule of practice, especially in such a case as Dr. Prewitt's, in the seventh month of pregnancy, when it is desirable to carry both mother and child through to a safe delivery. Perhaps bleeding would be of great service, but it is incorrect to say that, *be ause* there is albuminuria and congestion of the kidneys, there should be venesection. I do not see why, in any pregnant woman with congestion of the kidneys, there is any more reason for bleeding than in a case of Bright's disease without pregnancy. Dr. Prewitt's case does not present symptoms of acute nephritis even, and he says he found but one or two casts. There may have been a temporary local congestion in one or both of the kidneys. The amount of urine excreted seems to have been sufficient. He does not know the constituents of the urine other than albumen. It is well known, beyond all question of doubt, that albumen presents itself in the urine under a large variety of pathological conditions; and, if any albumen exists, it is a result, not a cause of renal congestion. If there is acute renal congestion, there will be blood in the urine. But that is not a basis of treatment, and we never can arrive, except empirically, at any proper basis of treatment in these cases, until we have arrived at a more thorough knowledge of the patho-logical and etiological conditions, and these are points that both Dr. Bauduy and myself attempted to treat of in our papers. The grounds that we both took were similar, not entirely denying the fact that parenchymatous nephritis was a common condition, but denying that, as a general rule, it was *the cause* of convulsive seizures. As to the subject being exhausted, I think we are but at the threshold; the very first men are studying it now with the utmost intensity, and I do not think a satisfactory conclusion has yet been reached. The probability is that these convulsions are due to a variety of causes. I am satisfied that Bright's disease never, or very rarely, causes convulsions, except in the later stages of the disease, or in acute forms in which not only the kidneys are congested, but the inflammation has extended to such a degree of functional destruction that there is a central neuropathic condition set up; and blood letting will not relieve that form of inflammation, and it never has been claimed that it would, and that condition does not constantly exist in puerperal eclampsia. I want to hear very much the expression of opinion from the other members of the society, on the real subject broached; that is the pathology, not the treatment, which is now empirical to a great extent.

Dr. Maughs: I did not mean to say that the society had left no further researches possible in this case. I remember though that it was discussed, and that I commended Dr. Moses' paper, giving the Traube-Rosenstein theory upon the subject. Rosenstein held that this is anemia, but anemia dependent upon hyperemia. Dr. Bauduy, in his paper, gave an exposition of the neuropathological theories of this question. He advocated the view that a large element of it is neurotic. I differ with Dr. Moses that a large number of cases are from nephritis. I think it is a very different thing. In this case, it is entirely different; it is simply a renal congestion, produced as is thought by most authorities, by pressure of the uterus, and a corrobation of this view is that it occurs most frequently in primiparæ, where there is great pressure from the uterus, keeping up a state of congestion, and interrupting the return of the circulation, thus producing albuminuria. We bleed in these cases, not so much to relieve the condition of the kidneys directly, as to depurate the blood. In the Traube-Rosenstein theory, blood-letting finds it advocacy, and in every other theory. If it be true that it relieves the local hyperemia, the anemia and the tension upon the brain, *relieves the anemia*, and removes the poisonous matter from the blood that produces the congestion, blood letting finds an advocacy even in the very nature of the pathology given by the neuropathologists. Now there is, perhaps, no subject under discussion in the world at the present day, that has received the universal sanction of any one remedy, that puerperal convulsions have. All over the civilized world, the lancet is advocated.

Twenty years ago, when we had to bleed a patient, we bled a certain amount, and if that did not relieve, we bled again. But now, owing to the blessing of chloroform, we are not driven to that necessity. We bleed them freely, while under the influence of chloroform, and move their bowels, and after they are moved, treat them with morphine; controlling the convulsions by breaking their force, depurating the blood, and relieving the hyperemia by the lancet. As it is not advisable to keep them unduly under the influence of chloroform, we give them hypodermic injections of morphine. I defy any man to bring forward any mode of treatment which has the same unanimity of approval throughout the world as this has. But it may not be necessary to bleed always. I simply would treat them with-saline cathartics, jarborandi, etc., unless the convulsions were imminent.

Jaborandi has the effect of sweating the patient profusely. If the symptoms were serious, I would give the same advice, as I gave Dr. Boisliniere some time ago in the case of a primipara in the eight month, to bring on premature labor, since in all probability, if he waited, she would have another and another convulsion, and he had better bring on an immediate delivery, so that the congestion of the kidneys would subside. A remarkable and wonderful proof of the pathology as far as the albuminuria is concerned, is given in the fact of the rapid recovery of those patients after confinement.

We all know that paients in the last extremity, after these convulsions, do recover rapidly after confinement. Convulsions do not produce albuminuria, and albuminuria does not necessarily produce convulsions, but they run very closely together, and a woman who has had that condition of the blood is almost certain to have albumen in the urine. I thought Dr. Bauduy's paper was a most able exposition of the subject as it stands at the present time.

Dr. Bauduy: I merely wish to say as regards what Dr. Barrett said in reference to the oxytoxic effect of jaborandi, that I think it is very probable, from the fact, that it is diverted in its action to the genito-urinary system to a great extent; and I am satisfied from experience, and I see it stated by Dr. Bartholow in his book, that it is the most powerful aphrodisiac remedy known at the present day. Therefore, it is not improbable that it may exert some oxytoxic effect, because what would have an effect upon the erotic powers might also have some oxytoxic effect.

I think the subject of my paper has not been touched upon in this particular respect. I carefully avoided any allusion to the treatment, because I did not think it had anything to do with the pathology of the matter. According to my ideas on the subject there are two forms, the hyperemic and the anemic. If it be the hyperemic form, the remedies should be antiphlogistic, blood-letting, purgatives, etc. In other words, all cases of pueperal convulsions are not to be treated from the same standpoint, and not in an empirical way. The point I made was this: that in these cases, there is almost always a neuropathic condition and that women who have eclamptic seizures are very likely to have other nervous disorders. The next point I made was on blood letting. There is a retention of excrementitious matters in the blood, poisoning the nervous system, in an accumulation of the blood corpuscles, and therefore, the blood is changed almost *in toto* adding to the irritability of the already overloaded system. Barnes states that one of the peculiarities in these cases, is the extreme irritability of the cerebrospinal axis. It is known that when the blood retains certain excrementitious matters that should not be retaintained, or other poisons, specific, or otherwise, vegetable, mineral or animal, it is known that such conditions lead to a state of irritability of the nervous system, and if such an irritability naturally exists, it becomes augmented. Another point taken was, that these conditions had the effect to act directly on the sympathetic system of nerves; and that that condition was irritation of the vaso motor nerves, not being positively paralyzed, but being in a state that leads to a spasmodic condition of the blood vessels, producing a more or less persistent anemia in the cerebral circulation. I alluded to the fact that chloroform acted well, in all probability, in both the anemic condition existing in these cases—the chloroform relieved by producing a relaxation of these blood vessels, and that where, on the other hand, there was a hyperemic condition, it is not unreasonable to suppose the chloroform produced it in consequence of its well known ordinary action. One of the dangers in the administration of chloroform is the superinduction of a fatal syncope; therefore, in the opposite class of cases, chloroform relieves the state of the cerebral vessels which precipitates syncope. Therefore, whatever, the condition, chloroform relieves it.

Dr. Prewitt: In this connection the treatment is of special interest, because I want to get my patient through. But so far as the condition of the kidneys is concerned, I venture to say that there never was a case of albuminuria in which there was not congestion of the kidneys, either active or passive, and the amount of albumen is always in direct proportion to the amount of congestion of the kidneys. It may be passive congestion or an active one. Now, in this case, it is certainly not an acute desquamative nephritis—such as we have following scarlet fever, in which there is an acute erythematous disease—but there is congestion, a decided congestion, of the kidney. It may be that bleeding would relieve that temporarily, and doubtless it would to a certain extent; but would it relieve the condition so that we can hope to get the woman through for the next two months, by purgatives and other things, inasmuch as the very cause of this condition is present all the time? Now, as to its being a neurosis, if Dr. Bauduy means by that, that the convulsions are simply the result of certain nerve conditions, brought about by other conditions, we will agree in that. But if he means to say that the convulsions in these cases are due to primary changes in the nerve centres, I cannot think so. I look upon it as very much the condition of things we have in poisoning from strychnia for instance. The convulsive movements in poisoning from strychnia certainly have their origin in the spinal cord, but we would hardly call it neurosis. And in these cases there is poisoning of the blood, and in cases of convulsions it is the cause of hyperemia of the brain I take it, and not puerperal hyperemia. In fact. in some cases I suspect it is almost exclusively that; in other cases there probably is hyperemia of the brain. But I must confess that I do not exactly understand the sense in which the doctor uses the term neurosis, as applied to this effect; but this is a side issue. I cannot think that albuminuria is a mere coincidence in many of these cases. I am satisfied that in a great majority of cases of puerperal convulsions, this condition is the cause of the convulsions.

Dr. Bauduy: I want to say very distinctly that I certainly did not mean that there are structural changes which are not palpable. The very term implies that if there are structural changes they are palpale. But what I meant to say was, that in these cases there is an unstable condition of the nervous element, that there is a neurotic predisposition to those eclamptic attacks, and that one woman predisposed to eclampsia in consequence of her hereditary tendencies, and transmutation of nervous disease, will develop an eclamptic attack under the excitement of pregnancy, and that another woman similarly placed, without this neurotic disposition, will not have an attack of eclampsia. Just as in men, one man will succumb to an attack of insanity under certain circumstances, if he is predisposed to it, whilst another without any such predisposition, to it, will resist the tendency. Now, in regard to this particular matter, it is hard to define the terms. We simply mean that it is some hidden condition of the nervous system that tends to develop these neuropathic affections. If Dr. Prewitt asks me what this unstable condition is, I am free to admit that I do not consider it is explicable. But the great point in these cases, is that women develop these terrible attacks in consequence of an inherent predisposition on their part, just as they have a tendency to the development of cerebral hemorrhages and a tendency to the development of puerperal mania, and if they do not have puerperal mania at some period of their lives, they suffer from hysterical attacks, epileptic attacks, or choreic manifestations. Just so in children it is claimed that those who have a preternatural mobility of the nervous system, children of neurotic parents may, from the slightest causes, worms, indigestion, etc., be thrown into convulsions; another child will go through precisely the same influences, and have no convulsions. There is just one more point with which I take issue with Dr. Prewitt, and that is this: Dr. Prewitt has said these conditions of nephritis and of kidney lesion are undoubtedly and unequivocally causes of puerperal eclampsia, in the majority of cases. That, from an etiological point of view, it is a most important matter. I claim as opposed to that theory, that if in two or three cases of puerperal eclampsia, it is proved that the kidneys are healthy, your theory is entirely annihilated.

Dr. Boisliniere: Dr. Bauduy holds that this condition of the nervous system is inexplicable. This is a mere flight of the fancy of your neuropathologists. A woman will have an attack of eclampsia and recover completely, and never have any insanity or nervous symptoms whatever. In the most perfect health she will have an attack of eclampsia and recover completely. It is sufficient to go back to the changes in the blood brought about by this kidney congestion. This also gives explanation why Dr. Barrett's suggestion is good, why everything that eliminates is good—it is because they are depurating remedies. I do not say that all these cases are hyperemic, but blood-letting will answer admirably in those cases where there is evidence of congestion—when the woman is black in the face, etc.

Dr. Bauduy: I feel compelled to make a reply to Dr. Boisliniere. In the first place, Dr. Boisliniere has misunderstood me. As a matter of course, I meant to say that this inherent tendency is something which is not palpable. It is something that the anatomist's scalpel cannot display; it is something which is manifested by a neurotic tendency; of that fact I do not suppose there can be any possible doubt. But, even with regard to these very cases, in which Dr. Boisliniere so absolutely declares that because a woman recovers she has not a neurotic constitution, I would be able to accept that statement, if he could tell me, whether, in the number of cases he has treated in his large experience, he has inquired minutely into their history, and whether he knows the history of the mothers, grandmothers and sisters of the patients, and whether he knows what their condition was prior to his treatment, and what it was after his treatment; whether he is certain that they have not died in insane asylums, or that they have not had choreic, epileptic or appoplectic manifestations. Those are very important points, so important that they cannot be excluded from an investigation of this kind. Then, in the second place, as regards the treatment: In all cases of puerperal eclampsia, even in cases of anemia of the brain, venesection may be a very valuable remedy, because we know that collateral hyperemias may exist, and the threatening symptoms of collateral hyperemia are the very symptoms which are often relieved by venesection, even in cases of extensive anemia of the brain.

Dr. Maughs: It seems that neuropathies are on the rampage at the present day, and that every thing is to be diverted into the neuropathic line. Now, if Dr. Bauduy means by inherent tendency, in eclampsia, that a woman has a nervous system, I agree with him. I do not believe it possible to produce eclampsia in a woman who has no nervous system. If he means that it depends upon a peculiar unstability of the nervous system, I also agree with him. You might just as well expect to throw a bull into spasms by tickling his ear, as to produce eclampsia, or a corresponding condition of the brain in a red man of the prairies. It has been known for a long time that women have a nervous system, and that, like a child's nervous system, it is more mobile and irritable than in the ordinary male subject. It has also been long contended that in pregnancy there is a sort of excitability intended to meet the wants of parturition. We have, in the pregnant condition, a very ready means of accounting for puerperal eclampsia, and I dare say you will search in vain in the records of medical science to the days of Hippocrates, to prove a connection between eclampsia in the daughter and in the mother, and it has never occurred to any of them that their mother had eclampsia. It is not hereditary, don't run in the line of families, and never did. Some women, of course, would be more inclined to it than others. Phlegmatic women of coarse, nervous systems would not be as susceptible to it as women of refined and delicate nervous systems. But further than that, I think that Dr. Bauduy can prove nothing. Rosenstein's theory will not stand in many cases; it has often been demonstrated to be true, and it has often been demonstrated to be not true.

A pregnant woman has not only thrown into her system the necreobiotic substance of her own system, but also that of her child's. If the kidneys be sluggish in their action, the blood very quickly becomes loaded with poisonous matters, producing a state of the system very readily producing eclampsia.

Dr. Bauduy: I wish to correct Dr. Maughs in this, that I never made the absurd statement that puerperal eclampsia was hereditary. I simply referred to the theory of the transmutation of nervous diseases.—*St. Louis Courier of Medicine.*

